# New agri-environmental measures have a direct effect on wildlife and economy on conventional agricultural land

**DOI:** 10.7717/peerj.15000

**Published:** 2023-03-21

**Authors:** Petr Marada, Jan Cukor, Michal Kuběnka, Rostislav Linda, Zdeněk Vacek, Stanislav Vacek

**Affiliations:** 1 Mendel University of Agriculture and Forestry, Brno, Czech Republic; 2 Forestry and Game Management Research Institute, Prague, Czech Republic; 3 Czech University of Life Sciences, Prague, Czech Republic; 4 University of Pardubice, Pardubice, Czech Republic

**Keywords:** Agroecology, Agricultural Economics, Multiscale ecosystem services, *Capreolus capreolus*, *Lepus europaeus*

## Abstract

The objective of this article is to evaluate economic profits along with return on investment and also the impact of newly designed agri-environmental measures (AEM) on the presence of bioindicator species—European hare and roe deer—in comparison to conventionally cultivated agricultural land. The abundance of European hare was, on average, 4.5–6.7 times higher on AEM compared to the standard agricultural regime and 3.5–6.4 times higher in the case of roe deer in 2020 and 2021. From an economic point of view, the highest incomes were found for extensive orchard alleys and standard conventional crops—wheat and rapeseed rotation. The cash flow from extensive orchard was 4.3 times larger and wheat and rapeseed were 3.5 times larger than from the clover grass mixture. Moreover, the lowest value of operational expenses was found in the case of extensive orchard alleys. The payback period ranged from 16.02 years (wheat and rapeseed rotation) to 53.6 years (clover grass mixture). It is crucial not to assess the economic parameters separately but optimize them with sustainable wildlife management and other benefits that provide ecological and efficient directions of AEM for future generations. However, the performed economic analysis highlights the significantly lower incomes of most components of AEM. We see a principal issue of AEMs usage in the lack of strong incentives for farmers to maximize conservation outcomes. Therefore, the AEMs are often placed in locations with presumed low agriculture profit, which is often related to insignificant conservation effects. Thus, the incomparable AEMs profitability compared to conventional agriculture has to be reflected by the agricultural policy at the European Union level and subsidy policy of particular member states.

## Introduction

The European landscape has been under agricultural management for millennia (Batáry et al., 2015) and presently, agricultural land covers nearly half of the total land area of the European Union (Groeneveld et al., 2019). Agricultural land dominates land use not only in the EU, but the entire Earth as well (Stoate et al., 2001; Stoate et al., 2009; Tanentzap et al., 2015). This long history of land transformation and agricultural utilization has resulted in accelerating environmental difficulties over the last few decades (Stoate et al., 2001). The negative consequences of intensive (conventional) agriculture on the environment are revealed in many processes, including the impact on climate change through greenhouse gas emissions, water pollution, air pollution, and degradation of the soil and ecological processes as well (Kleijn et al., 2009; Maia, Miyamoto & Garcia, 2018; Hristov et al., 2020; Was et al., 2021). The use of pesticides and mineral fertilizers in conventional agriculture often harms the environment through water pollution and eutrophication, and soil degradation (Foley et al., 2011; Tilman et al., 2011; Charles, Godfray & Garnett, 2014). Altogether, the intensification of agriculture, along with the declining number of cultivated species of crops, have led to a dramatic biodiversity loss and an overall homogenization of the landscape into open agricultural land (Bianchi, Booij & Tscharntke, 2006; Kalda, Kalda & Liira, 2015; Marada et al., 2019). This trend of intensive agricultural production associated with habitat changes goes hand in hand with the decline of wildlife that lives on farmland. A significant decline was observed in bird species, especially in ground-nesting endangered species such as grey partridge (*Perdix perdix* L.), northern lapwing (*Vanellus vanellus* L.), or corn crake (*Crex crex* L.), and other farmland birds (Stowe et al., 1993; Tyler, Green & Casey, 1998; Báldi & Faragó, 2007; Petrovan, Ward & Wheeler, 2013; Kamp et al., 2015; Weterings et al., 2016; Traba & Morales, 2019; Šálek et al., 2021; Šálek & Mayer, 2022). However, the decline was also documented for small and medium-sized mammals (Pavliska et al., 2018; La Haye et al., 2020; Šálek, Václav & Sedláček, 2020), herpetofauna (Barrett & Guyer, 2008; Thompson, Nowakowski & Donnelly, 2016) and insects (Beketov et al., 2013; Hausmann et al., 2022).

European hare (*Lepus europaeus* Pallas) is one of the formerly common species that has experienced a population decline mainly due to increasing agricultural intensification (Smith, Jennings & Harris, 2005; Cukor et al., 2018; Pavliska et al., 2018; Schai-Braun et al., 2020). In heavily farmed landscapes, it is difficult for a European hare to find suitable shelter, not only from predators but also from adverse climatic conditions. This problem is especially true for juveniles (Hackländer, Arnold & Ruf, 2002; Zellweger-Fischer, Kéry & Pasinelli, 2011), where the absence of suitable shelter can impact essential physiological processes, such as thermoregulation which results in negative energy balances (Hackländer, Arnold & Ruf, 2002). Therefore, the European hare is considered one of the bioindicator species of agricultural land quality (Pfister et al., 2002; Vávrová et al., 2003; Ujhegyi et al., 2021). On the other hand, there are a few wildlife species that have successfully adapted to current agricultural land. Wild ungulates are one example that has seen a rapid population increase in recent decades (Baltzinger et al., 2016; Valente et al., 2020; Carpio, Apollonio & Acevedo, 2020). Roe deer (*Capreolus capreolus* L.) belongs to the most widespread native ungulate species in European farmland with stable or increasing population density according to specific regions (Carpio, Apollonio & Acevedo, 2020; Hinojo et al., 2022). However, agricultural intensification poses a risk to roe deer fawns due to mortality during fodder harvest (Jarnemo, 2002; Steen et al., 2012; Cukor et al., 2019a; Cukor et al., 2019b) because along with red fox (*Vulpes vulpes* L.) predation, farming activities are given as the main cause of roe deer fawn mortality (Jarnemo, 2002; Jarnemo et al., 2004).

Organic farming is often promoted as significantly more environmentally friendly (for wildlife) when compared to conventional agricultural practices (Mäder et al., 2002; Hobbs, Sayre & Gupta, 2008; Tuck et al., 2014; Gomiero, 2021). In recent decades, there has been a gradual evaluation of the impact of established organic farming practices on agroecosystems compared to conventional agriculture (Hole et al., 2005). This comparison of the development of organic and conventional agriculture production in Europe is described in numerous studies (Mäder et al., 2007; Hobbs, Sayre & Gupta, 2008; Mäder & Berner, 2012; Tuck et al., 2014; Le Campion et al., 2020). Similarly, several papers are already available that compare the institutional and legislative aspects of these different forms of farming (*e.g.*, Haring, 2004) or by comparing the economic efficiency of conventional and organic farms (Offermann & Nieberg, 2000). However, from a conservation perspective, it is crucial to assess the environmental impacts, focusing on specific wildlife species known to have experienced long-term declines in their distribution and abundance as a result of previously implemented agricultural intensification (European Commission, 2019).

Organic farming is defined as management without synthetic inputs, *i.e.,* without synthetic pesticides and mineral fertilizers (Seufert, Ramankutty & Foley, 2012; Reganold & Wachter, 2016). Positive impacts on biodiversity result from specific management practices, especially biological conservation methods in organic farming systems that are either not used at all or rarely used in mainstream conventional agricultural systems (Clough, Kruess & Tscharntke, 2007; Paull, 2011; Mäder & Berner, 2012; Eschen et al., 2012). Several studies show that organic farming increases biodiversity and reduces environmental impact (Mäder et al., 2002; Tuck et al., 2014; Bender, Wagg & Heijden, 2016). Organic farming favors practices based on minimum tillage, permanent soil cover with phytomass and postharvest crop residues or support crops, allowing for appropriate crop rotations (Pittelkow et al., 2015). Moreover, the positive impact on soil quality and soil biota was proven (Hobbs, Sayre & Gupta, 2008; Köhl, Oehl & Van der Heijden, 2014; Briones & Schmidt, 2017). Thus, reducing the intensity of agricultural land use can directly affect the survival of the aforementioned species. Particularly important is the application of organic farming practices with the inclusion of selected agri-environmental measures focusing on supporting wildlife species. Those measures are known to have experienced long-term declines in their distribution and abundance as a result of previously implemented agricultural intensification (European Commission, 2019).

Based on these reasons, the EU Common Agricultural Policy (CAP) is currently under revision. The European Commission’s suggestions highlight a tighter focus on the environment and climate objectives than previously (European Commission, 2019; Hasler et al., 2019). Therefore, the agri-environmental measures (AEM) have come to constitute the main policy instrument to address environmental objectives within the European Union’s CAP (Bartolini & Vergamini, 2019). AEM are voluntary incentive measures aiming to steer the adoption of environmentally friendly practices (Gatto, Mozzato & Defrancesco, 2019), enhance species richness, and provide ecosystem services for the support of endangered animal species (mostly ground-nesting birds) and biodiversity of agricultural land (Boetzl et al., 2019). However, despite a 20-year application window and large budgetary shares allocated by EU member states, several studies demonstrate lower-than-expected environmental impacts especially on biodiversity richness (Aviron et al., 2007; Bartolini & Vergamini, 2019; Ujhegyi et al., 2021).

Interesting debates focusing on a balance between the ecological and economic benefits of AEM are continuing (Kleijn et al., 2006; Quillérou, Fraser & Fraser, 2011; Riley, 2016; Boetzl et al., 2019). The economic side/rationale significantly influences farmers’ decision to take up AEM (Defrancesco et al., 2008; Burton & Paragahawewa, 2011). From an economic point of view, the inefficiency of the AEM is linked to the violation of the principle of fiscal equivalence. Possible efficiency gains are sacrificed to distributional motives, as expressed, for example, in the EU cohesion policy (Uthes & Matzdorf, 2013). AEM funding comes mainly from the EU and the rest from national and regional governments. This interweaving of disparate decision-making scales has implications for the choice of AEM by different Member States (Kirschke et al., 2007). Farmers’ willingness to engage in agri-environmental policies also depends on the level of education, farm size, previous experience of farmers, and amicable relationships (Vanslembrouck, Van Huylenbroeck & Verbeke, 2002; Defrancesco et al., 2008).

Therefore, this article focuses on a complex description of AEM benefits concerning wildlife and the agricultural economy. The partial aims are to (i) evaluate the impact of newly designed AEM consisting of the combination of wildflower strip, extensive orchard alley, and clover grass mixture on the presence of European hare—a bioindicator species of agricultural land and roe deer, which is a common ungulate species of agroecosystems, in comparison to conventionally cultivated agricultural land; (ii) evaluate the economic profits of AEM compared to crops commonly cultivated in Central Europe; and to (iii) describe the economic return of investment in agricultural land in the case of the implementation of AEM in comparison with conventional agricultural management.

## Materials & Methods

### Study site

**Figure 1:**
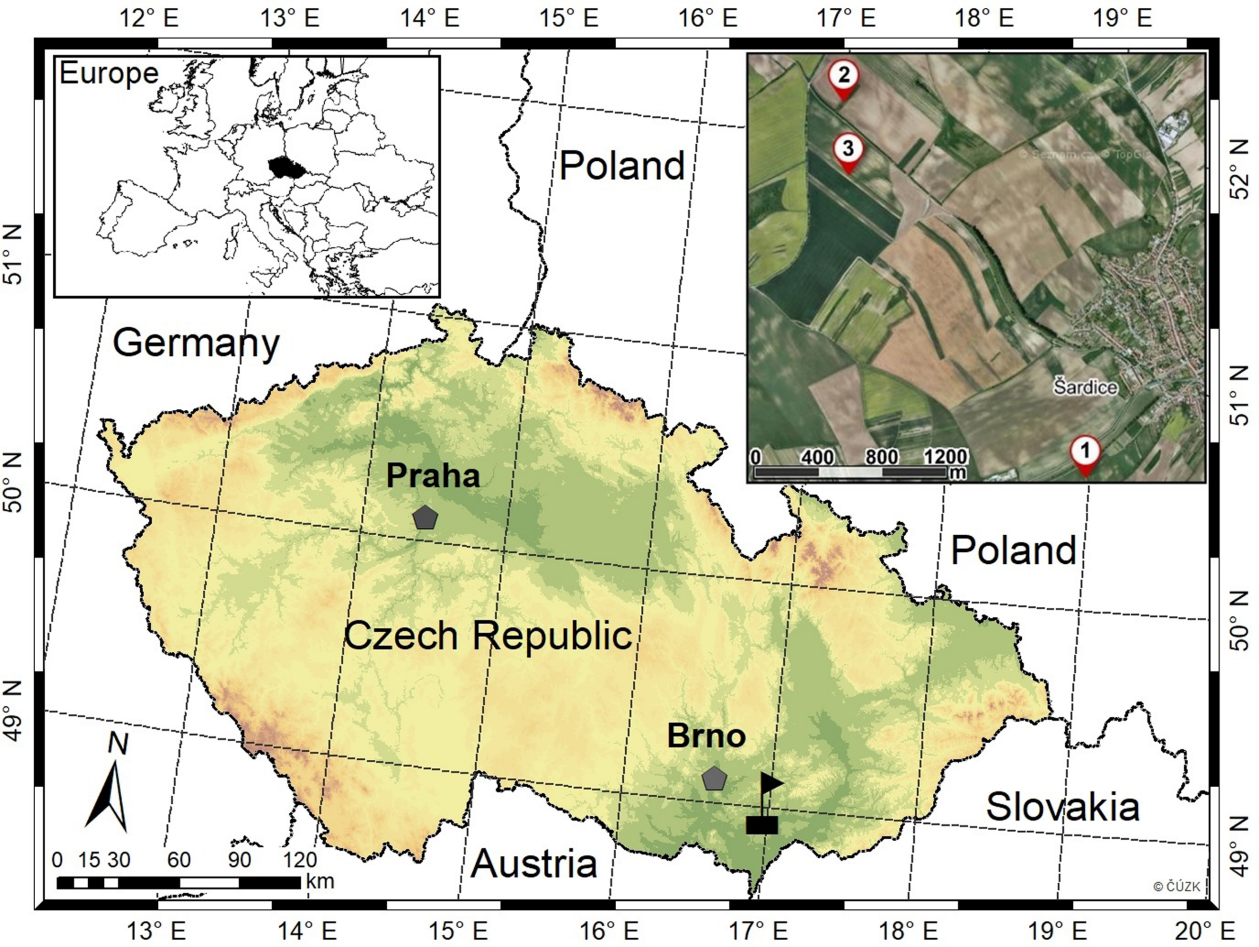
Localization of permanent research plots (1–3) within the hunting district of Šardice in southeastern Moravia, Czech Republic.

The study was performed in three locations (permanent research plots–PRPs) in the southeastern part of Moravia, Czech Republic, in the cadastral territory of Šardice (Fig. 1). The mean annual precipitation covering the wider area is approximately 509 mm, and the mean annual temperature fluctuates around 9.2 °C (CHMI, 2022). The length of the vegetation season reaches up to 170 days, with the highest mean temperature in July (19.3 °C) and the highest mean sum of precipitation also in July (66 mm). The lowest mean temperature is observed in January (−1.9 °C), and the lowest mean sum of precipitation in November (9.1 mm). There are ca. 37 days with snow cover in the area. The bedrock in the location consists mostly of marl, and the soil is identified as Chernozem (World Reference Base for Soil Resources, 2022) with a variable layer of humus.

From the aspect of hunting management, all three PRPs (size of ca. 1 ha) are situated in the hunting district of Šardice, with acreage of about 1833 ha. The basic details of PRPs are described in Table 1. The agricultural land for the given hunting district covers 1666 ha (90.9%), forests cover 83 ha (4.5%), water area comprises 8 ha (0.4%), and other areas (*e.g.*, roadways, recreational areas, urban greenery, field roads, etc.) make up 76 ha (4.1%). Altogether, approximately 330 ha (19.8% of agricultural land) are managed in the ecological regime of agriculture (mostly extensive orchard alleys, wildflower strips, etc.). In the hunting district, there is only roe deer as a representative of the ungulate game species. The average number of roe deer is 156 counted individuals (the average number for the period 2019–2020) counted by local game managers by direct counting method in non-vegetation season (before reproduction), according to Mayle, Peace & Gill (1999). Every year, 61 individuals are hunted per year (the average number for the period 2019–2020 in the entire hunting district; 3.3/100 ha). The European hare is another game species present in the hunting district. There were 228 individuals hunted per year (the average number for the time period 2019–2020 in the entire hunting district; 12.4/100 ha).

**Table 1:** Overview of basic characteristics of permanent research plots.

**Research plot ID**	**GPS coordinates**	**Acreage** (monitored area/whole plot; m^2^)	**Altitude** (m)	**Neighboring crop** (west side; 2020/2021)	**Agri-environmental measures**			**Neighboring crop** (east side; 2020/2021)
					Clover grass mixture (m^2^)	Extensive orchard alley (m^2^)	Wildflower strip (m^2^)	
PRP 1	48°57′27.30 ″N 17°01′10.11 ″E	10,161/ 13,785	197	rapeseed/wheat (5.5 ha)	3387	3387	3387	wheat/rapeseed (28.2 ha)
PRP 2	48°58′44.32 ″N 16°59′54.74 ″E	8,545/ 8,545	229	rapeseed/wheat (27 ha)	2848	2848	2848	wheat/rapeseed (24 ha)
PRP 3	48°58′29.22 ″N 16°59′56.29 ″E	10,000/ 31,612	225	rapeseed/wheat (15.5 ha)	3333	3333	3333	wheat/rapeseed (4 ha)

### Description of agri-environmental measures (AEM)

**Figure 2:**
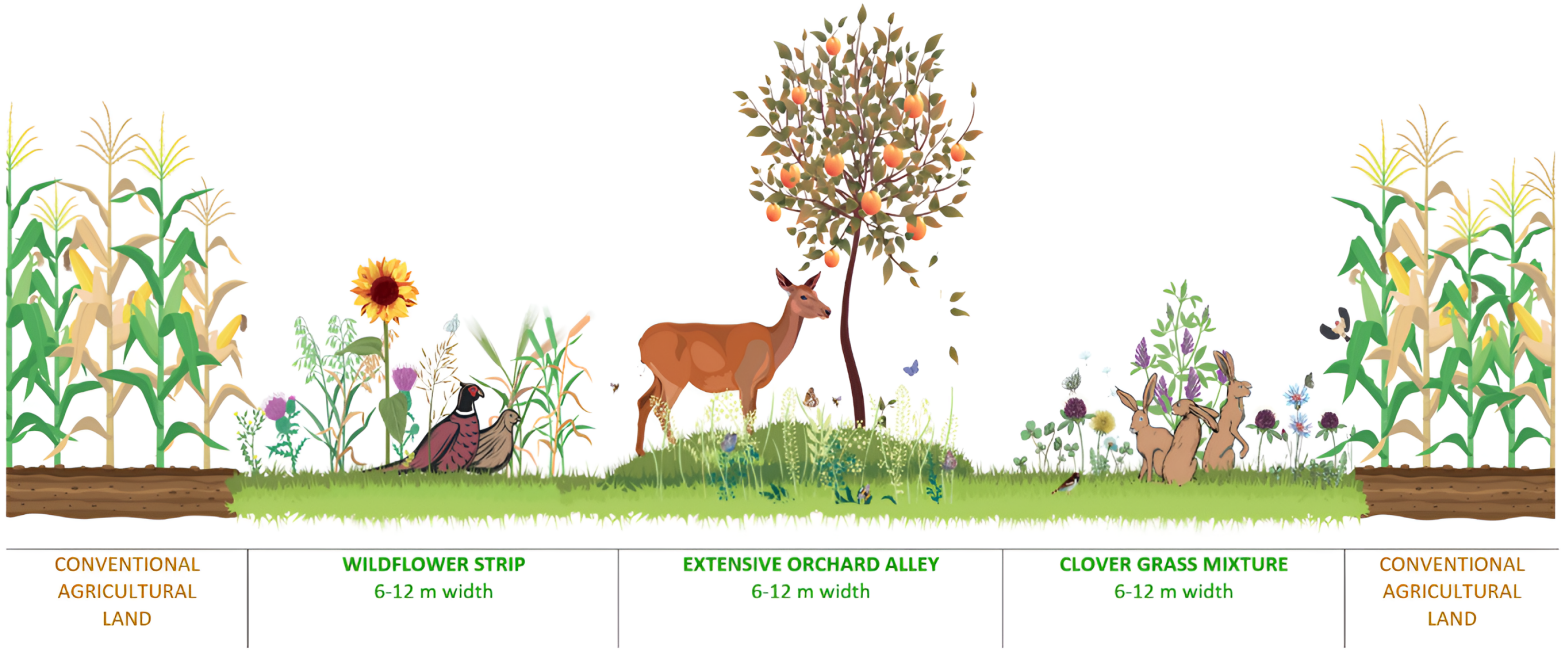
New design of agri-environmental measures.

Both the ecological effects and economy of AEM were evaluated on three PRPs. The schematic design of AEM is visualized in the following picture (Fig. 2). Every agri-environmental measure has approximately the same acreage of clover grass mixture and an extensive orchard alley with grassy undergrowth and a wildflower strip. In the extensive orchard alley were planted common fruit trees like European pear (*Pyrus communis* L.), apple tree (*Malus domestica* Borkh.), apricot (*Prunus armenica* L.), sour cherry (*Prunus cerasus* L.), European plum (*Prunus domestica* L.), or sweet cherry (*Prunus avium* L.) in numbers of 110–160 trees per hectare. The wildflower strip was sown by 105.8 kg of seeds/ha and the clover grass mixture was sown by 25 kg of seeds/ha. The wildflower strip consisted of common oat (*Avena sativa* L.; 65 kg), brown-corn millet (*Panicum miliaceum* L.; 15 kg), common buckwheat (*Fagopyrum esculentum*, Moench.; 15 kg), wild cabbage (*Brassica oleracea* L. var*. Acephala*; 0.8 kg), lacy phacelia (*Phacelia tanacetifolia*, Benth.; 5 kg), and white lupin (*Lupinus albus* L.; 5 kg). The clover grass mixture was sown with perennial grass (*Festuca pratensis*, Huds.; 3.75 kg), bulbous oat grass (*Arrhenatherum alatius ne elarius a doplnit* L.; 1.25 kg), timothy grass (*Phleum pratense* L.; 0.75 kg), smooth meadow-grass (*Poa pratensis* L.; 2.5 kg), creeping red fescue (*Festuca rubra* L.; 2.5 kg), alfalfa (*Medicago sativa* L.; 3.75 kg), common kidney vetch (*Anthyllis vulneraria* L.; 1.25 kg), crimson clover (*Trifolium incarnatum* L.; 1.75 kg), red clover (*Trifolium pratense* L.; 2.5 kg), common bird’s-foot trefoil (*Lotus corniculatus* L.; 1.25 kg), and Dutch clover (*Trifolium repens* L.; 3.75 kg). The AEM were located in the standard, conventionally managed farmland with normal field block acreage. The particular crops on neighboring field blocks are mentioned in Table 1 for every year of monitoring. The description and costs of agricultural operations which were done for the cultivation of clover grass mixture, wildflower strip, and extensive orchard alley are described in File S1.

### Wildlife monitoring

The utilization of AEM by European hare and roe deer was monitored in the AEM and the neighboring agricultural land by direct personal monitoring with a thermal camera equipped with a range-finder (Pulsar Accolade). Monitoring was conducted during the morning (05:00–06:00 AM) and evening hours (08:00–09:00 PM) for the optimum thermal contrast between monitored animals and the surrounding landscape. By using the Pulsar Accolade, it is possible to monitor the whole area of PRPs with the control plots in the surrounding landscape (described in Table 1). The numbers of observed individuals were averaged and recalculated to the acreage of 1 ha for a clear comparison. The European hares were personally monitored every morning and evening for 30 days in April 2020 and 2021 when the vegetation cover was low, and the animals were well recognizable by a thermal camera. The roe deer monitoring was done for 31 days in May 2020 and 2021. This time period overlaps with the season when roe deer fawns are born (Christen, Janko & Rehnus, 2018).

### AEM economic analysis

Financial analyses were calculated on differences between costs and incomes in AEM and conventional agriculture. The amount of subsidy was calculated according to Government Regulation No. 75/2015 on the conditions for implementing agri-environmental climate measures of the EU subsidy system. The costs of agriculture measures were calculated according to the average prices common in 2020–2021. Items of costs and incomes according to particular measures are listed in Files S1 and S2.

Moreover, the financial returns were calculated for different potential agricultural uses of the land. The selected land use variants are associated with different investment costs of the land purchase. The authors assume that when purchasing land, the price will neither be influenced by the prospective agricultural management, nor by the land valuation classification. Therefore, normal market prices of agricultural land of varied utilization were used for the analyses. Land prices were calculated on the basis of the data from the largest real estate portal in the Czech Republic, Sreality.cz, as the median of market prices of land in the category “field”, “orchard” and “meadow” in the South Moravian Region in late April 2022. For the calculation of a usual price, land plots with an area of more than 0.5 ha, intended for farming and not adjacent to residential districts were used. Land prices, operational income, operational expenses, and other values were converted from CZK to EUR at the exchange rate of CZK 25.625/EUR as of July 27, 2021, according to the Czech National Bank (2021). In the case of conventional crops (wheat, rapeseed) and wildflower strips, the median of the total number of 102 sale offers were considered. In the case of clover grass mixture, the median of 12 available offers were considered, and for the extensive orchard alley, the median of 26 available offers. The land prices were: A Wildflower strip 15,220 EUR/ha, B Extensive Orchard Alley 25,366 EUR/ha, C Clover Grass Mixture 14,634 EUR/ha, D Wheat 15,220 EUR/ha, E Rapeseed 15,220 EUR/ha. Cash flow from land management is the difference between operational income and expenses in individual years. The income from the sale of cultivated commodities, which was part of operational income, was valued according to the commodity prices stated by the Czech Statistical Office for 2021 (CSO, 2022). The costing of operational expenses corresponds to the sum of the financial valuation of the individual agrotechnical operations required to ensure the sowing (including the seed), treatment, and harvesting of each crop. These inputs are costed out at the current prices, in the form of services. An average price, determined by demand from three farms in the study area (according to Files S1 and S2), was considered. The cash flow structure is constant over a 5-year period, and recurring over a 5-year interval.

Some of the subsequently applied indicators for assessing economic efficiency take into account the time value through discounts. There will be three levels of interest rate used for discounting, namely:

Rf—risk-free interest rate—this will be derived, as recommended by theory, from the risk-free yield on government bonds corresponding to the life of the bond. A 15-year investment period, derived from 5-year and 10-year government bond yields, assuming a linear increase during its appreciation, as there are currently no Czech government bonds with a 15-year maturity on the capital market. According to the Kurzy.cz web portal (Kurcy.cz, 2021), the yield of the basket of government bonds with an average remaining maturity of 5 years (June 30, 2021) was 1.55% p.a., and with an average remaining maturity of 10 years 1.67% p.a. The yield of government bonds with a 15-year maturity was calculated assuming a linear relationship between the yield and the number of years of maturity—it yielded 1.79% p.a.

Rd—(cost of debt rate) the interest rate on debt financing—determined as the average variable interest rate on new mortgages. This was 2.14% p.a. as of May 31, 2021 (Kurcy.cz, 2021).

WACC—the weighted average cost of capital is a value of an agriculture holding’s cost of capital in which equity and debt capital are proportionately weighted. In NACE (NACE means Nomenclature Générale des Activités économiques dans les Communautés Européennes” and it is the statistical classification of economic activities in the EU.) A: Agriculture, Forestry and Fisheries, the Ministry of Industry and Trade (MPO.CZ) reported WACC costs of 11.74%.

### Evaluating the economic efficiency of investments

The economic efficiency of the investment in the land with regard to its management will be assessed using the following indicators: Internal rate of return (IRR), net present value (NPV), profitability index (PI), payback period (PP), and discounted payback period (DPP). These selected methods will effectively evaluate whether the investment in the land is profitable for each scenario of subsequent management and which of the variants of agricultural management (variants A to D and E rotation) is the most economically attractive. These indicators are commonly used in various industries, including agriculture, forestry, or ecology, to assess the efficiency of investments (see, *e.g.*, Kang et al., 2020; Sharma & Irmak, 2020). PP (Eq. (1)) *versus* DPP (Eq. (2)) does not take into account the time value of money. The PP indicator shows how many years it takes for the investor to recover the initial investment through operating income from the investment. The PP must be shorter than the life of the investment.

(1) \begin{eqnarray*}II={\mathop{\sum \nolimits }\nolimits }_{i=1}^{PP}C{F}_{t}\end{eqnarray*}

where: PP—Payback Period, CF—Future Cash Flows, II—Initial Investment, t—time period.

DPP operates by discounting future cash flows and recognizing the time value of money. The discount rate can be set at an rf, rd, and WACC. Again, the critical threshold is the life of the investment, which the DPP must not exceed, and when multiple options are selected, the investment with the shortest discounted payback period is the most appropriate. The indicator appears as follows.

(2) \begin{eqnarray*}II={\mathop{\sum \nolimits }\nolimits }_{i=1}^{DPP} \frac{C{F}_{t}}{(1+d)^{t}} \end{eqnarray*}

where: DPP—Dynamic Payback Period, CF—Future Cash Flows, d—discount rate of return that could be earned in alternative investments, II—Initial Investment, t—time period.

Net present value (NPV; Eq. (3)) calculates the current total value of a future cash flow stream. According to the NPV, an acceptable investment is the one that has a positive net present value, and the best investment that has the maximum NPV. The NPV is the difference between the accumulation of discounted net cash flows in each year of the life of the investment and the capital expenditure at the beginning of the investment. Calculation of NPV; see, *e.g.*, Gren & Amuakwa-Mensah (2020) or Sharma & Irmak (2020).

(3) \begin{eqnarray*}NPV=PVCF-II={\mathop{\sum \nolimits }\nolimits }_{i=1}^{n} \frac{C{F}_{t}}{(1+d)^{t}} -II\end{eqnarray*}

where: NPV—Net Present Value, PVCF—Present Value of Future Cash Flows, CF—Future Cash Flows, II—Initial Investment, d—discount rate of return that could be earned in alternative investments, t—time period, n—total number of time periods.

However, the NPV indicator is only one of several potential dynamic indicators used to evaluate the acceptability of an investment. The NPV indicator can be complemented by the Profitability Index PI if the size of the investment differs between investment options, as in this case. In the case of an acceptable investment, the resulting PI index (Eq. (4)) reaches a minimum value ≥ 1; see, *e.g.*, Ellabban & Alassi (2019) or Yoomak, Patcharoen & Ngaopitakkul (2019).

(4) \begin{eqnarray*}PI= \frac{PVCF}{II} = \frac{\sum _{i=1}^{n} \frac{C{F}_{t}}{(1+d)^{t}} }{II} \end{eqnarray*}

where: PI—Profitability Index, PVCF—Present Value of Future Cash Flows, CF—Future Cash Flows, II—Initial Investment, d—discount rate of return that could be earned in alternative investments, t—time period, n—total number of time periods.

The IRR is the discount rate at which the NPV equals 0. When evaluating an investment on the basis of IRR, the principle is that an investment is acceptable only if the value achieved for this indicator is higher than the appreciation of the risk-free investment rf or equal to and higher than the cost of capital rd or WACC (Hazen & Magni, 2021).

(5) \begin{eqnarray*}{\mathop{\sum \nolimits }\nolimits }_{i=1}^{n} \frac{C{F}_{t}}{(1+IRR)^{t}} -II=0\end{eqnarray*}

where: IRR—Internal Rate of Return, CF—Future Cash Flows, II—Initial Investment, d—discount rate of return that could be earned in alternative investments, t—time period, n—total number of time periods.

### Data analysis

For comparing differences between European hare and roe deer in 2020 and 2021, the Wilcoxon rank-sum test was employed. All statistical procedures were conducted in R software (R Core Team, 2018). The significance level was set to *α* = 0.05. Principal component analysis (PCA) was carried out in CANOCO 5 software (Lai, 2013) to assess the relationship between wildlife density and economic parameters of both AEM and conventional agriculture. Before the analysis, the data was standardized and centralized. The results of PCA were illustrated by an ordination diagram. Data from individual PRPs were merged for statistical evaluation because there was no significant difference between them.

## Results

**Figure 3:**
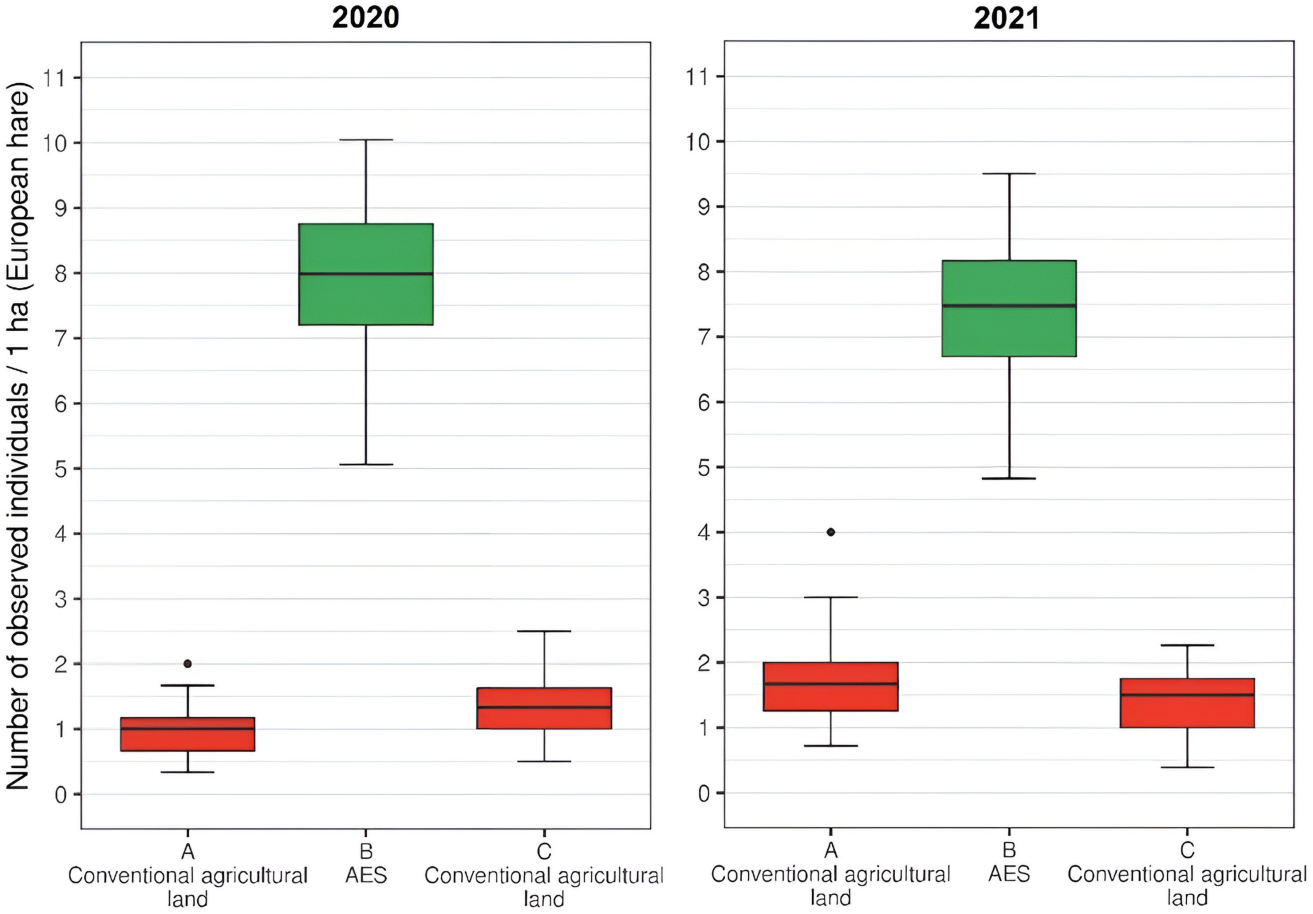
The numbers of detected European hares on agricultural land (per ha) with conventional practice and agri-environmental measures in 2020 and 2021.

**Figure 4:**
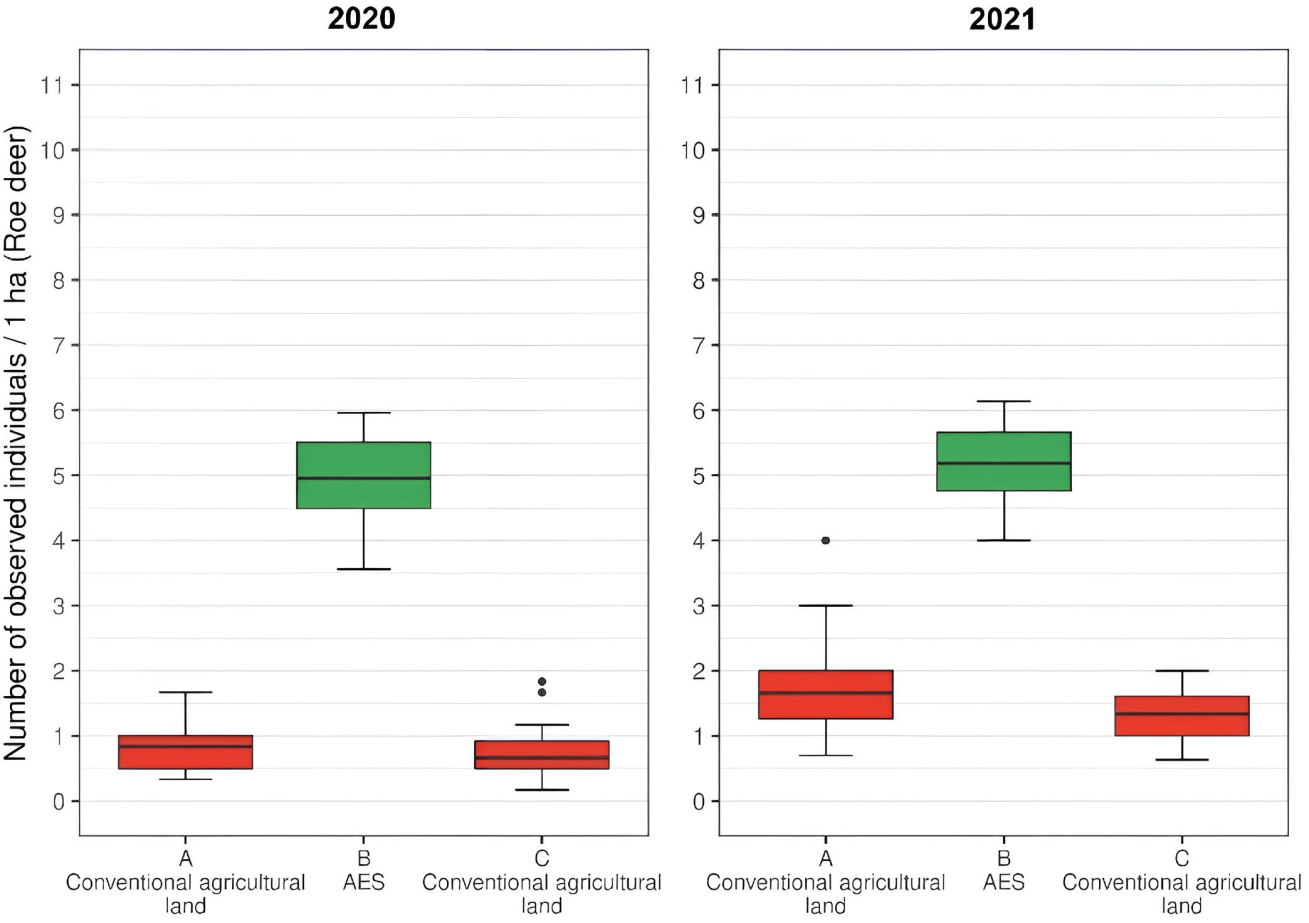
The numbers of detected roe deer on agricultural land (per ha) with conventional practice and agri-environmental measures in 2020 and 2021.

The testing of differences between the number of observed individuals on a landscape with AEM and neighboring landscape with conventional agricultural practice was performed *via* a paired-sample Wilcoxon rank-sum test. The tests showed significant differences (*p* < 0.001 in all cases) for both study species (European hare and roe deer; see Figs. 3 and 4) in both studied years. In the case of the European hare, on average, 1.18 individuals per ha (SD = 0.54) were observed on the land with conventional agricultural practices over a defined time, while the average of 7.86 (SD = 1.66) individuals per ha were observed on the land with an agri-environmental measure in 2020. The next year, 1.68 hares per ha (SD = 0.59) were observed for standard agricultural regime and 7.50 individuals per ha (SD = 1.60) in AEM (Fig. 3). In the case of roe deer, a significantly higher number of individuals were observed in AEM (mean = 4.89, SD = 1.73) compared with a standard agricultural regime (mean = 0.77, SD = 0.43) in 2020. One year later, a higher number of deer was observed for both cases, while conventional agricultural practices once again showed a significantly (*p* < 0.001) lower number of deer (mean = 1.50, SD = 0.51) compared to AEM (mean = 5.21, SD = 1.39; Fig. 4).

### Economic advantage of using your own land

The selected land use variants differ in their income (revenue), expenditure (costs), and therefore, cash flow (net income) in each year of management. Most types of costs repeat each year at the same amount (var. A Wildflower strip, B Extensive Orchard Alley, D Wheat, E Rapeseed). Only clover grass mixture has higher costs in the first year, while in the following years of the 5-year management cycle, the costs remain the same (see Table 2). Considering the need to rotate the rapeseed and wheat crops, these two crops will alternate on the plot. Therefore, their revenues, costs, and cash flows will be calculated by averaging the two.

With regard to the assessed revenue parameter, the highest values were found for the extensive orchard alley (see Table 2). Net income (cash flow) from extensive orchard alley is four times higher than from clover grass mixture, and cash flow from wheat and rapeseed is nearly three times higher than from clover grass mixture, which ranks last (we assume that the harvested forage will be sold as feed).

The lowest value of operational costs (EUR 134) was found in the case of the extensive orchard alley management. The calculation considered an established orchard that is already fully fruiting. A relatively comparable operational expenditure was found for the wildflower strip (EUR 259) and clover grass mixture (EUR 284). These three farming variants differ significantly from the other two, in terms of costs (wheat EUR 639; rapeseed EUR 805; mean EUR 722). The operational costs have a wider variance compared to the level of income. If we compare the annual expenditure on the extensive orchard alley (EUR 134) and on rapeseed cultivation (EUR 805), we find a difference of six times their amount, in the case of wheat—five times their amount. In terms of income, the maximum difference in revenue between extensive orchard alley and clover grass mixture is approximately three times (EUR 1685 wheat and rapeseed *vs.* EUR 558 clover grass mixture). Consequently, the calculated “cash flow” value corresponds to the highest attractiveness in the case of extensive orchard alley (EUR 1190) wheat and rapeseed cultivation (EUR 963), wildflower strip (EUR 673), clover grass mixture (EUR 274), as shown in Table 2.

**Table 2:** Ranking in terms of average annual revenue, costs, and cash flow (in EUR).

**Ranking**	**Revenue^a^**		Costs^a^		Cash flow^a^	
1	DE Wheat & Rapeseed	1685	B Extensive Orchard Alley	134	B Extensive Orchard Alley	1190
2	B Extensive Orchard Alley	1324	A Wildflower Strip	259	DE Wheat & Rapeseed	963
3	A Wildflower Strip	932	C Clover Grass Mixture	284	A Wildflower Strip	673
4	C Clover Grass Mixture	558	DE Wheat & Rapeseed	722	C Clover Grass Mixture	274

**Notes.**

a Revenue maximization, expenditure minimization, cash flow maximization.

### Economic advantage of land investment

Investment land has the lowest cost for growing clover grass mixture (EUR 14,634/ha), while the most expensive is the acquisition of an established orchard (EUR 25,366/ha). Investment in land for A Wildflower strip, D Wheat, and E Rapeseed is comparable to the purchase price of land, at an average of 15,220 EUR/ha. Table 3 shows that, in terms of the PP indicator, the fastest return on investment in agricultural land over 16 years was found in the case of sowing the land with wheat and rapeseed, followed by extensive orchard al-ley with a payback period of 20.32 years, and then the wildflower strip with a payback period of 22.61 years. Clover grass mixture clearly shows to be the worst value, with a return of almost 54 years.

**Table 3:** Payback period (PP) and discounted payback period (DPP).

	**A Wildflower Strip**	**B Extensive Orchard Alley**	**C Clover Grass Mixture**	**DE Wheat & Rapeseed**
Initial value (EUR)	15,220	25,366	14,634	15,220
PP (years)	22.61	20.32	53.58	16.02
DPP_rf_ (years)	28.06	26.09	>80	18.26
DPP_rd_ (years)	31.24	28.78	>80	19.92
DPP_WACC_ (years)	>80	>80	>80	>80

Considering the time value of money according to the DPP indicator and depending on the discount rate chosen (rf, rd, WACC), the payback period of the investment is extended. For both DPPrf and DPPrd, the ranking of the length of the investment payback period remains unchanged. The DPPrd values are higher than DPPrf, because rd is at the level of the capital cost when financed by a mortgage loan (2.14%), while rf reflects the alternative risk-free appreciation and is, therefore, lower (1.55%). DPPWACC maintains the previous ranking, with all investment payback periods at a discount rate of 11.74%, having a return above 80 years. Wheat and rapeseed (var. DE) are the most economically advantageous in terms of all of the indicators in Table 3. Extensive orchard alley (var. B) ranks second and wildflower strip (var. A) ranks third. Clover grass mixture (var. C) has the longest return period.

NPV values were also calculated, again with discount rates rf, rd, and WACC. In terms of NPVrf, wheat and rapeseed perform best and clover grass mixture worst. Generally, the land use variants do not achieve positive values over a time horizon of 15 years. This is due to the current high market value of land. In the case of the NPVrf calculation using 2020 land prices according to land suitability, all of the variants, reach negative NPVrf values. Wheat and rapeseed perform best with an NPVrf of EUR -3090.

NPVrd assesses whether the investment would be worthwhile if the land purchase were financed with a loan (Table 4). For all variants, the investment is not profitable with credit financing over a 15-year time horizon, as their NPVrd values <0. Again, wheat and rapeseed perform best with an NPV of EUR -3617. Using a discount rate of WACC, the NPV of all land use variants is negative. It follows that the farmer should use bank financing to finance such an investment and not use mixed corporate capital, as this will further reduce the NPV of the investment.

**Table 4:** Net present value (NPV) and profitability index (PI) over an investment time period of 15 years.

**A Wildflower Strip**																		
**YEAR**	**1**	**2**	**3**	**4**	**5**	**6**	**7**	**8**	**9**	**10**	**11**	**12**	**13**	**14**	**15**	**∑DCF**	**NPV**	PI
CF	673	673	673	673	673	673	673	673	673	673	673	673	673	673	673	x	x	x
rf	663	653	643	633	623	614	604	595	586	577	568	560	551	543	534	8947	-6273	0.5878
rd	659	645	632	618	605	593	580	568	556	545	533	522	511	500	490	8558	−6661	0.5623
WACC	602	539	482	432	386	346	309	277	248	222	198	178	159	142	127	4648	−10571	0.3054
**B Extensive Orchard Alley**																		
YEAR	1	2	3	4	5	6	7	8	9	10	11	12	13	14	15	∑DCF	NPV	PI
CF	1190	1190	1190	1190	1190	1190	1190	1190	1190	1190	1190	1190	1190	1190	1190	x	x	x
rf	1171	1154	1136	1119	1102	1085	1068	1052	1036	1020	1004	989	974	959	944	15,812	−9553	0.6234
rd	1165	1140	1116	1093	1070	1048	1026	1004	983	963	942	923	903	884	866	15,126	−10240	0.5963
WACC	1065	953	853	763	683	611	547	489	438	392	351	314	281	251	225	8216	−17150	0.3239
**C Clover Grass Mixture**																		
YEAR	1	2	3	4	5	6	7	8	9	10	11	12	13	14	15	∑DCF	NPV	PI
CF	125	311	311	311	311	125	311	311	311	311	125	311	311	311	311	x	x	x
rf	123	302	297	293	288	114	280	275	271	267	106	259	255	251	247	3628	−11006	0.2479
rd	122	298	292	286	280	110	268	263	257	252	99	242	236	231	227	3465	−11169	0.2368
WACC	112	249	223	200	179	64	143	128	115	103	37	82	74	66	59	1833	−12801	0.1253
**DE Wheat & Rapeseed**																		
YEAR	1	2	3	4	5	6	7	8	9	10	11	12	13	14	15	∑DCF	NPV	PI
CF	963	963	963	963	963	963	963	963	963	963	963	963	963	963	963	x	x	x
rf	899	885	871	858	845	832	819	807	795	782	770	759	747	736	724	12129	−3090	0.7969
rd	893	875	856	838	821	804	787	770	754	738	723	708	693	678	664	11603	−3617	0.7624
WACC	817	731	654	585	524	469	420	375	336	301	269	241	216	193	173	6302	−8918	0.4141

The PI indicator puts ∑DCF and the initial value into the ratio and thus takes into account the size of the initial investment, which can be crucial for the investor. PI >1 indicates that the discounted cash flow from the investment exceeds the initial investment. This is not the case for all variants, in terms of PIrf, as the discounted income will not reach the price of the purchased land even after 15 years. The closest to the value of 1 was the PIrf of wheat and rapeseed with 0.7969.

With credit financing (discount rate rd of 2021), the situation became worse, and all variants are below the economic acceptability threshold.

Taking into account the cost of equity and cost of debt through the discount rate at the WACC level (11.74%), none of the variants cross the critical threshold 1. Even in the case of wheat and rapeseed (DE) as the best economic appreciation of the land, the PIWACC value achieved was only 0.4141.

The IRR indicator estimates the annual rate of appreciation that an investment will generate each year over a given investment horizon. Table 5 clearly shows that the extreme increase in the market price of land in recent years has meant that Wildflower Strip (A), Extensive Orchard Alley (B), Clover Grass Mixture (C), and even Wheat and Rapeseed (DE) do not generate sufficient net income to ensure a return on investment over a 15-year time. For their discounted revenue to reach the value of the land investment, their discount rates would have to be negative. Specifically, clover grass mixture has a discount rate of −12.56%, extensive orchard alley, −4.11%, wildflower strip, −4.74%. Wheat and rapeseed generate the highest appreciation (−0.69%).

**Table 5:** Internal rate of return (IRR) over an investment time period of 15 years.

**IRR A Wildflower strip:**																		
**YEAR**	**1**	**2**	**3**	**4**	**5**	**6**	**7**	**8**	**9**	**10**	**11**	**12**	**13**	**14**	**15**	**∑CF**	**DCF**	IRR
CF	673	673	673	673	673	673	673	673	673	673	673	673	673	673	673	10096	x	
DCF	707	742	779	817	858	901	946	993	1042	1094	1148	1206	1266	1328	1395	x	15220	−4.74%
**IRR B Extensive Orchard Alley:**																		
YEAR	1	2	3	4	5	6	7	8	9	10	11	12	13	14	15	∑CF	DCF	IRR
CF	1190	1190	1190	1190	1190	1190	1190	1190	1190	1190	1190	1190	1190	1190	1190	17843	x	
DCF	1241	1294	1349	1407	1467	1530	1595	1664	1735	1809	1887	1967	2052	2139	2231	x	25366	−4.11%
**IRR C Clover Grass Mixture:**																		
YEAR	1	2	3	4	5	6	7	8	9	10	11	12	13	14	15	∑CF	DCF	IRR
CF	125	311	311	311	311	125	311	311	311	311	125	311	311	311	311	4112	x	
DCF	143	407	466	533	609	280	797	911	1042	1191	547	1558	1782	2038	2331	x	14634	−12.56%
**IRR DE Wheat & Rapeseed:**																		
YEAR	1	2	3	4	5	6	7	8	9	10	11	12	13	14	15	∑CF	DCF	IRR
CF	963	963	963	963	963	963	963	963	963	963	963	963	963	963	963	13687	x	
DCF	918	924	930	937	943	950	957	964	972	979	987	995	1004	1012	1021	x	14496	−0.69%

### Relationship between wildlife and economy

**Figure 5:**
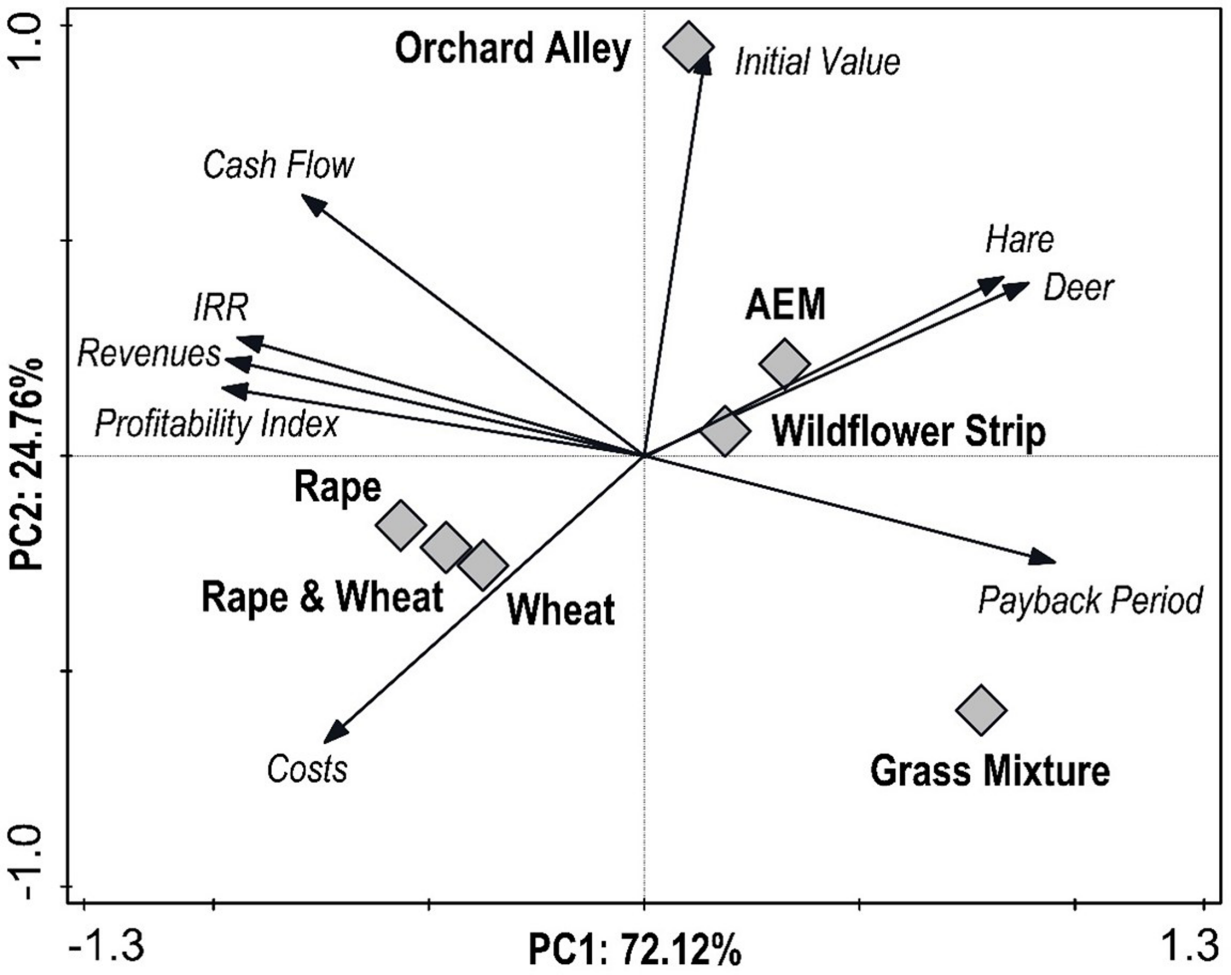
Ordination diagram showing the results of principal components analysis of the relationship between economic parameters (Payback Period, Expenditures, Profitability Index, Revenues, Internal Rate of Return, Cash Flow and Initial Value), wildlife abundance (Hare, Deer), and agricultural variants (Agro-environmental schemes—AES including Extensive Orchard Alley, Wildflower Strip, and Grass Mixture; Wheat & Rapeseed).

Principal component analysis results expressing the relationship between wildlife and economic parameters are presented in the form of an ordination diagram in Fig. 5. The first ordination axis represents 72.12%, the first two axes 96.88%, and the four axes combined account for 99.86% of data variability. The *x*-axis represents the profitability index and revenues. The *y*-axis represents the initial value. The internal rate of return was positively correlated with revenues, profitability index, and cash flow, while these parameters were negatively correlated with the payback period. The abundance of monitoring wildlife (hare and roe deer) was negatively correlated with all economic parameters except the initial value and payback period. The lowest difference between variants was observed for wheat and rape (and their combination), while the marks indicating grass mixture and orchard alley were very far from the others. Generally, high values of economic indicators were characteristic for wheat and rapeseed, while AEM, including grass mixture, wildflower strip, and extensive orchard alley, were typical for a high abundance of wildlife and payback period.

## Discussion

Effects of organic farming, AES and AEM are often measured on invertebrates and birds; mammals as indicator species are infrequently targets of such research (Ujhegyi et al., 2021). In our case, the European hare was selected because of its dramatic long-term population decline caused by changes in agricultural management (Smith et al., 2004; Smith, Jennings & Harris, 2005; Pavliska et al., 2018; Mori et al., 2022). Moreover, the hare is a suitable wildlife species for local agroecosystem improvement evaluation because of its relatively small home range sizes. The daily home ranges are in the range of units of hectares (Ullmann et al., 2020), and monthly home range sizes fluctuate in low tens of hectares (10–20 ha) (Rühe & Hohmann, 2004) in valuable agriculture areas with a smaller acreage of field blocks and high biodiversity, which is the case of our area of interest. This was also confirmed in a study from Germany and Denmark, where the mean of the daily home range acreage was 6.0 ha (median = 3.3 ha) with seasonal fluctuations and the lower acreage reducing agricultural field sizes (Mayer et al., 2019). The significant effects of landscape structure on home range size and population density of European hares were also documented previously (Smith et al., 2004; Pavliska et al., 2018; Mori et al., 2022), however, the direct effect of agricultural measures has only been verified in a few studies with conflicting results. A recent study from Hungary did not confirm a direct effect of AEM on supporting European hare population density (Ujhegyi et al., 2021). Similarly, a study from Italy did not find a benefit of organic farming on hare population density compared to conventionally managed landscapes (Santilli & Galardi, 2016). Moreover, Zellweger-Fischer, Kéry & Pasinelli (2011) describe that the number of set-asides/wildflower strips and European hare density were not related to arable or grassland sites. Contrastingly, in our case, hare abundance was found to be more than six times higher in modified agricultural land by agricultural measures, which by composition provides cover and food for the hare throughout the year. However, the high abundance found in research plots needs to be considered in the wider context of the area of interest, where approximately 20% of the agricultural landscape is managed in an organic regime. Similar findings were described by Meichtry-Stier et al. (2014), who confirmed that to attain desirable hare densities (and also strongly enhanced bird density), the required proportion of high-quality AES variants and seminatural habitat has to be at least 14% (Meichtry-Stier et al., 2014).

We have also confirmed a significant effect on roe deer presence which was ca. 3 times higher in AEM when compared to conventional agricultural land in both studied years. In general, roe deer have success-fully colonized open agricultural landscape for several decades (Gerard et al., 1995) and becomes locally overabundant (Valente et al., 2020; Carpio, Apollonio & Acevedo, 2020). The roe deer distribution on agricultural land is affected by landscape structure within the home range, whose mean monthly size is around 76.5 ha in European conditions (Morellet et al., 2013). The principal driver of home range size is spatial heterogeneity which goes hand in hand with fodder availability and cover sources. It affects the distribution and local roe deer population density (Said & Servanty, 2005; Morellet et al., 2013; Benjamin et al., 2022). The combination of AEM agricultural land utilization, including an extensive orchard alley, wildflower strip, and clover grass mixture provides sufficient cover and fodder sources throughout the year also after harvest of conventional agricultural crops. Moreover, the extensive management with a low rate of intervention of agricultural machinery, including fodder harvest, reduces the roe deer fawn mortality during the spring season, which is one of the most important reasons for roe deer mortality in agroecosystems (Jarnemo, 2002; Jarnemo et al., 2004). This positive environmental aspect therefore prevents and significantly reduces the damage caused to game during agroecosystem management.

It should be mentioned here that the acreage of one hectare with three repetitions must be reflected as a study limitation from the point of wildlife utilization of research plots. On the other hand, the differences in wildlife numbers were at a high level of significance. In addition, the research plot acreage was chosen with respect to the current common agricultural policy, where the minimum area in which a subsidy can be applied is 1 ha (CAP Strategic plan for the period, 2023-2027). Therefore, the confirmation of the significant impact of wildlife presence can be an important motivation for small landowners to apply CAP on their grounds.

The economic point of view is another important factor that decides if the AEM will be implemented on agricultural land. It has been shown that, in most cases, the current level of financial support is not enough to motivate farmers’ participation (Moon, 2013; Gatto, Mozzato & Defrancesco, 2019). However, in the case of AEM, the low frequency of agricultural interventions (and, of course, a low rate of expenditures) can be another motivating factor. In terms of the simple difference between revenues and expenditure each year on average for every five years of production, the extensive orchard alley appears to be the most suitable management method with a cash flow value of EUR 1190/year. The economic profit is related to organic fruit production, which does not involve the use of synthetic mineral fertilizers, pesticides, herbicides, fungicides, and growth regulators (DeEll & Prange, 1992; Van der Maas, 2007). In addition, increasing harvest efficiency (*e.g.*, mechanization of harvest cooperatives), increasing orchard value (*e.g.*, fruit processing options), and reducing labor costs by improving the availability of seasonal workers may add value in the future (Schönhart et al., 2011). The extensive orchard alley was followed by conventional agricultural crops. The profitability of wheat and rapeseed produced a cash flow of EUR 963/year. However, the profitability of conventional agricultural crops, especially rapeseed cultivated in the largest, large and medium-sized agricultural enterprises, was confirmed by previous studies (Seskena & Spogis, 2008). Moreover, specialist farmers with conventional agricultural crops and higher marginal land profitability (due to investment in fixed costs such as a combine harvester) are less willing to apply for AEM because the opportunity costs derived from changing cropping practices are higher (Espinosa-Goded, Barreiro-Hurlé & Dupraz, 2013). The other components of AEM produce significantly lower cash flow values. The wildflower strip was relatively close to conventional agricultural crops (EUR 673/year) due to the subsidy policy. In general, the concept of colorfields and blooming strips is profitable with either low labor costs, low tenure prices, or additional voluntary support by subsidy or sponsors (Paar, Röhricht & Schuler, 2008). In our study, the lowest cash flow values were found for clover grass mixture (EUR 274/year). On the other hand, farmers with livestock that can graze on this type of crop and thereby gain profit must be taken into account (Espinosa-Goded, Barreiro-Hurlé & Dupraz, 2013).

An economic evaluation was also carried out in the case of the purchase of agricultural land and its subsequent management. The price of the land represents an investment and the difference between operational income and expenditure related to the cash flow of crop production, which can be measured in various aspects against the investment in the land. However, given current land prices and their dramatic increase in recent years, investment in farmland does not appear as attractive. The reported values of the national average cost of agricultural land in 2020 are approximately EUR 10,000/ha (EUROSTAT, 2022). The average market price for 2021 was already EUR 15,220/ha for land suitable for growing wildflower strips and common agricultural commodities, and EUR 25,366/ha for orchard alleys. Despite the increase in recent years, farmland prices in the Czech Republic are around the EU average, while the highest prices of agricultural land were found in the Netherlands. The national average in 2020 was approximately EUR 70,000/ha (EUROSTAT, 2022). In terms of return on land price (PP and DPP coefficients), wheat and rapeseed are the most profitable crop, followed by extensive orchard alley and wildflower strip. Clover grass mixture has the worst values, hence the highest payback period values, due to the fact that the investment in land is slightly lower than for wildflower strips, wheat, and rapeseed, but the average annual cash flow for the other crops is 2.5 to 4 times higher. The economic return of an extensive orchard alley is fundamentally influenced by the purchase price of land, which is EUR 25,366/ha in the Czech Republic. These facts prove to have an impact on NPV but not PI. The NPV of all variants is negative, which means that after taking into account the expected appreciation at the level of rf, rd, or WACC, the land does not generate enough of a cash flow for the investment in the land to be recouped in a 15-year horizon. The best performing crop is again wheat and rapeseed, which, when discounted at the risk-free investment (rf) level, can generate a net income of 79.69% over 15 years (PI reaches 0.7969) compared to forage with a PIrf of 0.2479. According to the IRR indicator, the ranking of land use profitability is identical to the previous indicators.

In the case of the specific agri-environmental measures evaluated, which is composed of clover grass mixture, a wildflower strip, and an extensive orchard alley, it is necessary to consider both the economic and the direct impacts on the monitored species. In addition to supporting biodiversity, agri-environmental measures also allow for significant phytomass production. The proper function of grassed swales sown with forage mixtures allows for the retention of erosive sediment formed on neighboring intensively managed land. The increasing demand for agricultural phytomass resources and the pressure for a circular economy, *i.e.,* the use of eroded sediment as a soil forming substrate, will certainly increase the profitability of owners of such managed agri-environmental measures (Marada et al., 2021).

In terms of a general comparison of yields of conventional agriculture with organic farming, several studies have already been published. For example, a study by Ponisio et al. (2015), building on Seufert, Ramankutty & Foley (2012) and De Ponti, Rijk & Van Ittersum (2012), compared 1,071 paired yield observations across 115 studies and showed that organically managed fields have, on average, 19.2% lower yields compared to conventionally managed fields. It was also observed that the reduced yield between organic and conventional farming depended on the cropping pattern. Another meta analysis by Pittelkow et al. (2015) compared organic minimum tillage farming with conventional tillage farming, and observed that minimum tillage farming reduced yields by 5.7% on average compared to conventional tillage. These data suggest that the economic efficiency of organic farms is lower than conventional farms.

A major issue with AEMs usage is the lack of strong incentives for farmers to maximize conservation outcomes. Therefore, the AEMs are often placed by farmers to localities with low conservation effect (Sidemo-Holm, 2022) but in localities with low production potential from the point of conventional crops (Marada et al., 2021). Indeed, organic farming has the highest potential to support biodiversity cost-effectively in high-productive regions but is mainly employed in low-productive regions where the yield difference compared with conventional farming (*i.e.,* opportunity cost) is the lowest (Tuck et al., 2014; Sidemo-Holm, 2022). When the conservation outcomes are expected to be high, the most cost-effective policy solution would be to increase the payment to farmers to match the opportunity costs. The possible solution to motivate farmers to implement AEMs where and when they have the highest conservation potential, especially when this coincides with high opportunity costs, is to link payment rates with conservation outcomes (Sidemo-Holm, 2022). This has been shown to enable higher conservation outcomes given payment budgets (*e.g.*, Wuepper & Huber, 2021). Additionally, the ongoing development of organic practices, such as more efficient weed management, is contributing to closing the yield gap between organic and conventional farming (Röös et al., 2018). However, more intensive organic farming can compromise the environmental benefits of organic farming (Sidemo-Holm et al., 2021). It is, therefore, important that AEMs enable a combination of high yields and environmental benefits.

Based upon described information, it is evident that policy plays a crucial role in shaping the pro-environmental behavior of farmers, which includes such basic mechanisms as regulations (*e.g.*, limits on pesticide use) and economic instruments which pay farmers directly for adopting environmentally friendly practices (Tanentzap et al., 2015; Was et al., 2021; Sidemo-Holm, 2022). Agri-environmental schemes and measures are partly designed to address this. AES and AEM are a significant source for nature conservation funding within the European Union (EU) and the highest conservation expenditure in Europe (Batáry et al., 2015). The height of these expenditures will likely be preserved in future decades because the new agricultural policy for 2023–2027 guarantees that the 25% of all the budget for a common agricultural policy will be allocated for eco-measures such as organic farming, agroecology, carbon farming, etc. (European Commission, 2022). This document will be utilized by particular member states, including the Czech Republic, for their national strategic plans. Then the precise evaluation of agricultural practice based on new agricultural policy will be needed. The evaluation must be based on a multidisciplinary comparison of population dynamics of bioindicator species with impact on whole agroecosystems not only on a national level but in the context of the entire area of the European Union.

## Conclusion

The comprehensive evaluation of agri-environmental measures confirmed the direct impact of the measure on wildlife species on a local scale. In the case of the European hare, which is considered a bioindicator species, it was shown that the hare was over six times more abundant on AEM in the first year of study and more than four times more abundant in the second year compared to conventionally managed land. A similar effect was found for roe deer. In contrast, the economic evaluation showed a lower cash flow for most of the measures (wildflower strip and clover grass mixture) and a slower return on investment in farmland and subsequent extensive farming in the proposed system with a combination of wildflower strip and clover grass mixture, compared to the most commonly grown conventional crops. However, the benefits of AEM need to be considered in a broader context that takes into account highly desirable ecosystem services, especially anti-erosion, soil conservation, and the overall impact on biodiversity. From an economic point of view, AEM offers low input costs for farming and incomparably lower intensity of agrotechnical operations using agricultural machinery. This can be particularly important for smaller-area farmers with less availability of agricultural equipment.

##  Supplemental Information

10.7717/peerj.15000/supp-1 Data S1 Raw data

10.7717/peerj.15000/supp-2 Supplemental Information 2 Supplementary Tables

## Additional Information and Declarations

### Data Availability

The following information was supplied regarding data availability: The raw data are available in the Supplemental File.

## References

[ref-1] Aviron S, Jeanneret P, Schüpbach B, Herzog F (2007). Effects of agri-environmental measures, site and landscape conditions on butterfly diversity of Swiss grassland. Agriculture, Ecosystems and Environment.

[ref-2] Báldi A, Faragó S (2007). Long-term changes of farmland game populations in a post-socialist country (Hungary). Agriculture, Ecosystems and Environment.

[ref-3] Baltzinger M, Mårell A, Archaux F, Pérot T, Leterme F, Deconchat M (2016). Overabundant ungulates in French Sologne? Increasing red deer and wild boar pressure may not threaten woodland birds in mature forest stands. Basic and Applied Ecology.

[ref-4] Barrett K, Guyer C (2008). Differential responses of amphibians and reptiles in riparian and stream habitats to land use disturbances in western Georgia, USA. Biological conservation.

[ref-5] Bartolini F, Vergamini D (2019). Understanding the spatial agglomeration of participation in agri-environmental schemes: the case of the Tuscany Region. Sustainability.

[ref-6] Batáry P, Dicks LV, Kleijn D, Sutherland WJ (2015). The role of agri-environment schemes in conservation and environmental management. Conservation Biology.

[ref-7] Beketov MA, Kefford BJ, Schäfer RB, Liess M (2013). Pesticides reduce regional biodiversity of stream invertebrates. Proceedings of the National Academy of Sciences of the United States of America.

[ref-8] Bender SF, Wagg C, Van der Heijden MGA (2016). An underground revolution: biodiversity and soil ecological engineering for agricultural sustainability. Trends in Ecology and Evolution.

[ref-9] Benjamin CS, Uphus L, Lüpke M, Rojas-Botero S, Dhillon MS, Englmeier J, Fricke U, Ganuza C, Haensel M, Redlich S, Riebl R, Tobisch C, Uhler J, Zhang J, Menzel A, Peters W (2022). Modelling the relative abundance of roe deer (Capreolus capreolus L.) along a climate and land-use gradient. Animals.

[ref-10] Bianchi FJJA, Booij CJH, Tscharntke T (2006). Sustainable pest regulation in agricultural landscapes: a review on landscape composition. Biodiversity and Natural Pest Control.

[ref-11] Boetzl FA, Krimmer E, Krauss J, Steffan-Dewenter I (2019). Agri-environmental schemes promote ground-dwelling predators in adjacent oilseed rape fields: diversity, species traits and distance-decay functions. Journal of Applied Ecology.

[ref-12] Briones MJI, Schmidt O (2017). Conventional tillage decreases the abundance and biomass of earthworms and alters their community structure in a global meta-analysis. Global Change Biology.

[ref-13] Burton RJ, Paragahawewa UH (2011). Creating culturally sustainable agri-environmental schemes. Journal of Rural Studies.

[ref-14] CAP Strategic plan for the period (2023-2027). European Union.

[ref-15] Carpio AJ, Apollonio M, Acevedo P (2020). Wild ungulate overabundance in Europe: contexts, causes, monitoring and management recommendations. Mammal Review.

[ref-16] Charles H, Godfray H, Garnett T (2014). Food security and sustainable intensification. Philosophical Transactions of the Royal Society B: Biological Sciences.

[ref-17] Christen N, Janko C, Rehnus M (2018). The effect of environmental gradients on the bed site selection of roe deer (Capreolus capreolus). Mammal Research.

[ref-18] Clough Y, Kruess A, Tscharntke T (2007). Organic versus conventional arable farming systems: functional grouping helps understand staphylinid response. Agriculture, Ecosystems and Environment.

[ref-19] Cukor J, Bartoška J, Rohla J, Sova J, Machálek A (2019a). Use of aerial thermography to reduce mortality of roe deer fawns before harvest. PeerJ.

[ref-20] Cukor J, Havranek F, Linda R, Bukovjan K, Painter MS, Hart V (2018). First findings of brown hare (*lepus europaeus)* reintroduction in relation to seasonal impact. PLOS ONE.

[ref-21] Cukor J, Havránek F, Vacek Z, Bukovjan K, Podrázský V, Sharma RP (2019b). Roe deer (*capreolus capreolus*) mortality in relation to fodder harvest in agricultural landscape. Mammalia.

[ref-22] Czech Hydrometeorological Institute (CHMI) (2022). Czech Hydrometeorological Institute. https://www.chmi.cz/?l=enCzech.

[ref-23] Czech Statistical Office (CSO) (2022). Czech Statistical Office. https://www.czso.cz/csu/czso/home.

[ref-24] De Ponti T, Rijk B, Van Ittersum MK (2012). The crop yield gap between organic and conventional agriculture. Agricultural Systems.

[ref-25] DeEll JR, Prange RK (1992). Postharvest quality and sensory attributes of organically and conventionally grown apples. HortScience.

[ref-26] Defrancesco E, Gatto P, Runge F, Trestini S (2008). Factors affecting farmers’ participation in agri-environmental measures: a Northern Italian perspective. Journal of agricultural economics.

[ref-27] Ellabban O, Alassi A (2019). Integrated Economic Adoption Model for residential grid-connected photovoltaic systems: an Australian case study. Energy Reports.

[ref-28] Eschen R, Brook AJ, Maczey N, Bradbury A, Mayo A, Watts P, Buckingham D, Wheeler K, Peach WJ (2012). Effects of reduced grazing intensity on pasture vegetation and invertebrates. Agriculture, Ecosystems and Environment.

[ref-29] Espinosa-Goded M, Barreiro-Hurlé J, Dupraz P (2013). Identifying additional barriers in the adoption of agri-environmental schemes: the role of fixed costs. Land Use Policy.

[ref-30] Foley JA, Ramankutty N, Brauman KA, Cassidy ES, Gerber JS, Johnston M, Mueller ND, O’Connell C, Ray DK, West PC, Balzer C, Bennett EM, Carpenter SR, Hill J, Monfreda C, Polasky S, Rockström J, Sheehan J, Siebert S, Tilman D, Zaks DPM (2011). Solutions for a cultivated planet. Nature.

[ref-31] European Commission (2019). The post-2020 common agricultural policy: environmental benefits and simplification. https://knowledge4policy.ec.europa.eu/publication/post-2020-common-agricultural-policy-environmental-benefits-simplification_en.

[ref-32] European Commission (2022). The new common agricultural policy: 2023–27. https://agriculture.ec.europa.eu/common-agricultural-policy/cap-overview/new-cap-2023-27_en.

[ref-33] Gatto P, Mozzato D, Defrancesco E (2019). Analysing the role of factors affecting farmers’ decisions to continue with agri-environmental schemes from a temporal perspective. Environmental Science and Policy.

[ref-34] Gerard JF, Le Pendu Y, Maublanc ML, Vincent JP, Poulle ML, Cibien C (1995). Large group formation in European Roe deer: an adaptive feature?. Revue d Ecologie-la Terre et La Vie.

[ref-35] Gomiero T, Galankis CM (2021). Organic agriculture: impact on the environment and food quality. Environmental impact of agro-food industry and food consumption.

[ref-36] Gren IM, Amuakwa-Mensah F (2020). Multifunctional forestry and interaction with site quality. Forests.

[ref-37] Groeneveld AN, Peerlings JHM, Bakker MM, Polman NBP, Heijman WJM (2019). Effects on participation and biodiversity of reforming the implementation of agri-environmental schemes in the Netherlands. Ecological Complexity.

[ref-38] Hackländer K, Arnold W, Ruf T (2002). Postnatal development and thermoregulation in the precocial European hare (Lepus europaeus). Journal of Comparative Physiology B: Biochemical, Systemic, and Environmental Physiology.

[ref-39] Haring AM (2004). Organic farming and measures of European agricultural policy.

[ref-40] Hasler B, Czajkowski M, Elofsson K, Hansen LB, Konrad MT, HØ Nielsen, Niskanen O, Nõmmann T, Pedersen AB, Peterson K, Poltimäe H, Svensson TH, Zagórska K (2019). Farmers’ preferences for nutrient and climate-related agri-environmental schemes: a cross-country comparison. Ambio.

[ref-41] Hausmann A, Ulrich W, Segerer AH, Greifenstein T, Knubben J, Morinière J, Bozicevic V, Doczkal D, Günter M, Müller J, Habel JC (2022). Fluctuating insect diversity, abundance and biomass across agricultural landscapes. Scientific Reports.

[ref-42] Hazen G, Magni CA (2021). Average internal rate of return for risky projects. The Engineering Economist.

[ref-43] Hinojo A, Christe P, Moreno I, Hofmeister RJ, Dandliker G, Zimmermann F (2022). Estimating roe deer density using motion-sensitive cameras in Switzerland. Journal of Wildlife Management.

[ref-44] Hobbs PR, Sayre K, Gupta R (2008). The role of conservation agriculture in sustainable agriculture. Philosophical Transactions of the Royal Society B: Biological Sciences.

[ref-45] Hole DG, Perkins AJ, Wilson JD, Alexander IH, Grice PV, Evans AD (2005). Does organic farming benefit biodiversity?. Biological Conservation.

[ref-46] Hristov J, Clough Y, Sahlin U, Smith HG, Stjernman M, Olsson O, Sahrbacher A, Brady MV (2020). Impacts of the EU’s common agricultural policy Greening reform on agricultural development, biodiversity, and ecosystem services. Applied Economic Perspectives and Policy.

[ref-47] Jarnemo A (2002). Roe deer Capreolus capreolus fawns and mowing—Mortality rates and countermeasures. Wildlife Biology.

[ref-48] Jarnemo A, Liberg O, Lockowandt S, Olsson A, Wahlström K (2004). Predation by red fox on European roe deer fawns in relation to age, sex, and birth date. Canadian Journal of Zoology.

[ref-49] Kalda O, Kalda R, Liira J (2015). Multi-scale ecology of insectivorous bats in agricultural landscapes. Agriculture, Ecosystems and Environment.

[ref-50] Kamp J, Pelster A, Gaedicke L, Karthäuser J, Dieker P, Mantel K (2015). High nest survival and productivity of Northern Lapwings Vanellus vanellus breeding on urban brownfield sites. Journal of Ornithology.

[ref-51] Kang X, Lin R, O’Shea R, Deng C, Li L, Sun Y, Murphy JD (2020). A perspective on decarbonizing whiskey using renewable gaseous biofuel in a circular bioeconomy process. Journal of Cleaner Production.

[ref-52] Kirschke D, Hager A, Jechlitschka K, Wegener S (2007). Distortions in a multi-level co-financing system: the case of the agri-environmental programme of Saxony-Anhalt. Agrarwirtschaft.

[ref-53] Kleijn D, Baquero RA, Clough Y, Díaz M, De Esteban J, Fernández F, Gabriel D, Herzog F, Holzschuh A, Jöhl R, Knop E, Kruess A, Marshall EJP, Steffan-Dewenter I, Tscharntke T, Verhulst J, West TM, Yela JL (2006). Mixed biodiversity benefits of agri-environment schemes in five European countries. Ecology Letters.

[ref-54] Kleijn D, Kohler F, Báldi A, Batáry P, Concepción ED, Clough Y, Díaz M, Gabriel D, Holzschuh A, Knop E, Kovács A, Marshall EJP, Tscharntke T, Verhulst J (2009). On the relationship between farmland biodiversity and land-use intensity in Europe. Proceedings of the Royal Society B: Biological Sciences.

[ref-55] Köhl L, Oehl F, Van der Heijden MGA (2014). Agricultural practices indirectly influence plant productivity and ecosystem services through effects on soil biota.

[ref-56] Kurcy.cz (2021). The yield of the basket of government bonds. https://www.kurzy.cz/dluhopisy/vynos-uroky/.

[ref-57] Lai J (2013). Canoco 5: a new version of an ecological multivariate data ordination program. Biodiversity Science.

[ref-58] Le Campion A, Oury FX, Heumez E, Rolland B (2020). Conventional versus organic farming systems: dissecting comparisons to improve cereal organic breeding strategies. Organic Agriculture.

[ref-59] La Haye MJ, Van Kats RJ, Müskens GJ, Hallmann CA, Jongejans E (2020). Predation and survival in reintroduced populations of the common hamster Cricetus cricetus in the Netherlands. Mammalian Biology.

[ref-60] Mäder P, Berner A (2012). Development of reduced tillage systems in organic farming in Europe. Renewable Agriculture and Food Systems.

[ref-61] Mäder P, Fließbach A, Dubois D, Gunst L, Fried P, Niggli U (2002). Soil fertility and biodiversity in organic farming.

[ref-62] Mäder P, Hahn D, Dubois D, Gunst L, Alföldi T, Bergmann H, Oehme M, Amadò R, Schneider H, Graf U, Velimirov A, Fließbach A, Niggli U (2007). Wheat quality in organic and conventional farming: results of a 21 year field experiment. Journal of the Science of Food and Agriculture.

[ref-63] Maia AG, Miyamoto BCB, Garcia JR (2018). Climate change and agriculture: do environmental preservation and ecosystem services matter?. Ecological Economics.

[ref-64] Marada P, Cukor J, Linda R, Vacek Z, Vacek S (2019). Extensive orchards in the agricultural landscape: effective protection aagainst fraying damage caused by roe deer. Sustainability.

[ref-65] Marada P, Mareček J, Krčálová E, Krajíček T, Lacina L, Horák I (2021). The circular economics of revitalization process of concentrated water runoff paths and retention reservoirs.

[ref-66] Mayer M, Ullmann W, Heinrich R, Fischer C, Blaum N, Sunde P (2019). Seasonal effects of habitat structure and water on the habitat selection and home range size of a mammal in agricultural landscapes. Landscape Ecology.

[ref-67] Mayle BA, Peace AJ, Gill MA (1999). How many deer? A field guide to estimating deer population size.

[ref-68] Meichtry-Stier KS, Jenny M, Zellweger-Fischer J, Birrer S (2014). Impact of landscape improvement by agri-environment scheme options on densities of characteristic farmland bird species and brown hare (Lepus europaeus). Agriculture, Ecosystems and Environment.

[ref-69] Moon K (2013). Conditional and resistant non-participation in market-based land management programs in Queensland, Australia. Land Use Policy.

[ref-70] Morellet N, Bonenfant C, Börger L, Ossi F, Cagnacci F, Heurich M, Kjellander P, Linnell JDC, Nicoloso S, Sustr P, Urbano F, Mysterud A (2013). Seasonality, weather and climate affect home range size in roe deer across a wide latitudinal gradient within Europe. Journal of Animal Ecology.

[ref-71] Mori E, Carbone R, Viviano A, Calosi M, Fattorini N (2022). Factors affecting spatiotemporal behaviour in the European brown hare *Lepus europaeus*: a meta-analysis. Mammal Review.

[ref-72] Offermann F, Nieberg H (2000). Economic performance of organic farms in Europe (Organic farming in Europe: economics and policy).

[ref-73] Paar P, Röhricht W, Schuler J (2008). Towards a planning support system for environmental management and agri-environmental measures—the Colorfields study. Journal of environmental management.

[ref-74] Paull J (2011). The uptake of organic agriculture: a decade of worldwide development. Journal of Social and Development Sciences.

[ref-75] Pavliska PL, Riegert J, Grill S, Šálek M (2018). The effect of landscape heterogeneity on population density and habitat preferences of the European hare (*Lepus europaeus*) in contrasting farmlands. Mammalian Biology.

[ref-76] Petrovan SO, Ward AI, Wheeler PM (2013). Habitat selection guiding agri-environment schemes for a farmland specialist, the brown hare. Animal Conservation.

[ref-77] Pfister HP, Kohli L, Kästli P, Birrer S (2002). Feldhase Schlussbericht 1991–2000.

[ref-78] Pittelkow CM, Liang X, Linquist BA, Van Groenigen LJ, Lee J, Lundy ME, Van Gestel N, Six J, Venterea RT, Van Kessel C (2015). Productivity limits and potentials of the principles of conservation agriculture. Nature.

[ref-79] Ponisio LC, M’gonigle LK, Mace KC, Palomino J, De Valpine P, Kremen C (2015). Diversification practices reduce organic to conventional yield gap. Proceedings of the Royal Society B: Biological Sciences.

[ref-80] Quillérou E, Fraser R, Fraser I (2011). Farmer compensation and its consequences for environmental benefit provision in the higher level stewardship scheme. Journal of Agricultural Economics.

[ref-81] R Core Team (2018). https://www.r-project.org.

[ref-82] Reganold JP, Wachter JM (2016). Organic agriculture in the twenty-first century. Nature Plants.

[ref-83] Riley M (2016). How does longer term participation in agri-environment schemes [re]shape farmers’ environmental dispositions and identities?. Land Use Policy.

[ref-84] Röös E, Mie A, Wivstad M, Salomon E, Johansson B, Gunnarsson S, Wallenbeck A, Hoffmann R, Nilsson U, Sundeberg C, Watson CA (2018). Risks and opportunities of increasing yields in organic farming. A review. Agronomy for Sustainable Development.

[ref-85] Rühe F, Hohmann U (2004). Seasonal locomotion and home-range characteristics of European hares (Lepus europaeus) in an arable region in central Germany. European Journal of Wildlife Research.

[ref-86] Šálek M, Kalinová K, Dankova R, Grill S, Zmihorski M (2021). Reduced diversity of farmland birds in homogenized agricultural landscape: a cross-border comparison over the former Iron Curtain. Agriculture Ecosystems & Environment.

[ref-87] Šálek M, Mayer M (2022). Farmstead modernization adversely affects farmland birds. Journal of Applied Ecology.

[ref-88] Šálek M, Václav R, Sedláček F (2020). Uncropped habitats under power pylons are overlooked refuges for small mammals in agricultural landscapes. Agriculture Ecosystems & Environment.

[ref-89] Said S, Servanty S (2005). The influence of landscape structure on female roe deer home-range size. Landscape Ecology.

[ref-90] Santilli F, Galardi L (2016). Effect of habitat structure and type of farming on European hare (Lepus europaeus) abundance. Hystrix.

[ref-91] Schai-Braun SC, Ruf T, Klansek E, Arnold W, Hackländer K (2020). Positive effects of set-asides on European hare (Lepus europaeus) populations: Leverets benefit from an enhanced survival rate. Biological Conservation.

[ref-92] Schönhart M, Schauppenlehner T, Schmid E, Muhar A (2011). Analysing the maintenance and establishment of orchard meadows at farm and landscape levels applying a spatially explicit integrated modelling approach. Journal of Environmental Planning and Management.

[ref-93] Seskena I, Spogis K, Jakusonoka I (2008). Economical results of rape seeds in different economical size farms.

[ref-94] Seufert V, Ramankutty N, Foley JA (2012). Comparing the yields of organic and conventional agriculture. Nature.

[ref-95] Sharma V, Irmak S (2020). Economic comparisons of variable rate irrigation and fertigation with fixed (uniform) rate irrigation and fertigation and pre-plant fertilizer management for maize in three soils. Agricultural Water Management.

[ref-96] Sidemo-Holm W (2022). Time to incentivize cost-effective conservation in agricultural landscapes. Frontiers in Conservation Science.

[ref-97] Sidemo-Holm W, Carrié R, Ekroos J, Lindström SA, Smith HG (2021). Reduced crop density increases floral resources to pollinators without affecting crop yield in organic and conventional fields. Journal of Applied Ecology.

[ref-98] Smith RK, Jennings NV, Harris S (2005). A quantitative analysis of the abundance and demography of European hares Lepus europaeus in relation to habitat type. Mammal Review.

[ref-99] Smith RK, Jennings NV, Robinson A, Harris S, Smith RK (2004). Conservation of European hares Lepus europaeus in Britain: is increasing habitat heterogeneity in farmland the answer?. Journal of Applied Ecology.

[ref-100] Steen KA, Villa-Henriksen A, Therkildsen OR, Green O (2012). Automatic detection of animals in mowing operations using thermal cameras. Sensors.

[ref-101] Stoate C, Báldi A, Beja P, Boatman ND, Herzon I, Van Doorn A, De Snoo GR, Rakosy L, Ramwell C (2009). Ecological impacts of early 21st century agricultural change in Europe—a review. Journal of Environmental Management.

[ref-102] Stoate C, Boatman ND, Borralho RJ, Carvalho CR, De Snoo GR, Eden P (2001). Ecological impacts of arable intensification in Europe. Journal of Environmental Management.

[ref-103] Stowe TJ, Newton AV, Green RE, Mayes E (1993). The decline of the corncrake crex crex in britain and ireland in relation to habitat. The Journal of Applied Ecology.

[ref-104] Tanentzap AJ, Lamb A, Walker S, Farmer A (2015). Resolving conflicts between agriculture and the natural environment. PLOS Biology.

[ref-105] Thompson ME, Nowakowski AJ, Donnelly MA (2016). The importance of defining focal assemblages when evaluating amphibian and reptile responses to land use. Conservation Biology.

[ref-106] Tilman D, Balzer C, Hill J, Befort BL (2011). Global food demand and the sustainable intensification of agriculture. Proceedings of the National Academy of Sciences of the United States of America.

[ref-107] Traba J, Morales MB (2019). The decline of farmland birds in Spain is strongly associated to the loss of fallowland. Scientific Reports.

[ref-108] Tuck SL, Winqvist C, Mota F, Ahnström J, Turnbull LA, Bengtsson J (2014). Land-use intensity and the effects of organic farming on biodiversity: a hierarchical meta-analysis. Journal of Applied Ecology.

[ref-109] Tyler GA, Green RE, Casey C (1998). Survival and behaviour of Corncrake Crex crex chicks during the mowing of agricultural grassland. Bird Study.

[ref-110] Ujhegyi N, Keller N, Patkó L, Biró Z, Tóth B, Szemethy L (2021). Agri-environment schemes do not support brown hare populations due to inadequate scheme application. Acta Zoologica Academiae Scientiarum Hungaricae.

[ref-111] Ullmann W, Fischer C, Kramer-Schadt S, Pirhofer-Walzl K, Glemnitz M, Blaum N (2020). How do agricultural practices affect the movement behaviour of European brown hares (Lepus europaeus)?. Agriculture, Ecosystems and Environment.

[ref-112] Uthes S, Matzdorf B (2013). Studies on agri-environmental measures: a survey of the literature. Environmental Management.

[ref-113] Valente AM, Acevedo P, Figueiredo AM, Fonseca C, Torres RT (2020). Overabundant wild ungulate populations in Europe: management with consideration of socio-ecological consequences. Mammal Review.

[ref-114] Van der Maas MP (2007). Increasing high quality production of organically grown apples through a system’s approach including management of Vf scab resistance. Acta Horticulturae.

[ref-115] Vanslembrouck I, Van Huylenbroeck G, Verbeke W (2002). Determinants of the willingness of Belgian farmers to participate in agri-environmental measures. Journal of Agricultural Economics.

[ref-116] Vávrová M, Zlámalová Gargošová H, Šucman E, Večerek V, Kořínek P, Zukal J, Zejda J, Sebestiánová N, Kuvištová L (2003). Game animals and small terrestral mammals - sustainable bioindicators for the pollution assessment in agrarian ecosystems. Fresenius Environmental Bulletin.

[ref-117] Weterings MJA, Zaccaroni M, Van der Koore N, Zijlstra LM, Kuipers HJ, Van Langevelde F, Van Wieren SE (2016). Strong reactive movement response of the medium-sized European hare to elevated predation risk in short vegetation. Animal Behaviour.

[ref-118] Wąs A, Malak-Rawlikowska A, Zavalloni M, Viaggi D, Kobus P, Sulewski P (2021). In search of factors determining the participation of farmers in agri-environmental schemes—does only money matter in Poland?. Land Use Policy.

[ref-119] World Reference Base for Soil Resources (2022). International soil classification system for naming soil and creating legends for soil maps.

[ref-120] Wuepper D, Huber R (2021). Comparing effectiveness and return on investment of action-and results-based agri-environmental payments in Switzerland. American Journal of Agricultural Economics.

[ref-121] Yoomak S, Patcharoen T, Ngaopitakkul A (2019). Performance and economic evaluation of solar rooftop systems in different regions of Thailand. Sustainability.

[ref-122] Zellweger-Fischer J, Kéry M, Pasinelli G (2011). Population trends of brown hares in Switzerland: The role of land-use and ecological compensation areas. Biological Conservation.

