# Supplementary material for: New agri-environmental measures have a direct effect on wildlife and economy on conventional agricultural land
Source: PeerJ. 2023 Mar 21;11:e15000. doi: 10.7717/peerj.15000 (PMC10038087; doi:10.7717/peerj.15000)
Supplement: Supplementary file 1 [file peerj-11-15000-s002.docx]

Supplementary file S1. Costs of AEM and conventional agriculture.

Material	Wildflower strip (EUR ha -1)	Extensive orchard (EUR ha -1)	Clover grass mixture (EUR ha-1)	Winter wheat (EUR ha-1)	Rapeseed (EUR ha-1)
Seed	122.4	A landscape-forming orchard is already established or will be established with 100% subsidy support	161.8	43.1	76.3
Fertilizers	-	-	-	153.9	202.5
Pesticides	-	-	-	133.7	206.6
**Total**	**122.4**	**-**	**161.8**	**330.7**	**485.4**
**Agrotechnical operation***					
Stubble tillage	20.3	Mowing – 31.2 (ungrazed grass 1× in the spring, second cut in July)	4.1 (1× in 5 years 20.2)	16.2	16.2
Plowing	46.5	Levelling – 17.0	21.9 (1× in 5 years 109)	44.6	44.6
Preparation for sowing	20.3 (harrowing, dragging)	-	4.1 (1× in 5 years 20.2)	16.2 (soil compactor)	16.2 (soil compactor)
Sowing	36.4	-	7.3 (1× in 5 years 36.4)	32.4	32.4
Spraying (incl. import of water)	-	-	-	48.7	60.8
Fertilizing (incl. import)	-	Restorative pruning – 40.5	-	48.7	48.7
Harvest of commodities	-	Hay collection and baling – 52.2<br>Fruit picking & pick-your-own fruit	Hay collection and baling – 52.7	72.9	81.0
Transport of commodities	-	Bale loading and transport – 24.3<br>Fruit transport	Bale loading and transport – 24.3	20.3	12.2
Other (land inspection throughout the year)	8.1	8.1	7.8	8.1	8.1
**Total**	**136.6**	**134.0**	**122.2**	**308.1**	**320.2**

Notes: *The cost of the operation includes repairs and depreciation of machinery, direct wages and fuel; the higher cost of selected operations is due to farming a smaller area of the land block

Supplementary file S2. Incomes of AEM and conventional agriculture.

**Gross margin**	**Wildflower strip**	**Extensive orchard**	**Clover and grass mixture**	**Winter wheat**	**Rapeseed**
**Purchase price (EUR t-1)**	**not rated**	**Market price of hay – 89.4**<br>**Pick-your-own fruit price – 0.78 EUR kg-1**	**Market price of hay – 89.4**	**196.0**	**469.5**
**Yield (t ha-1)**	**not harvested**	**1.72 t of hay**<br>**1000 kg.ha-1 of fruit**	**1.72 t of hay**	**6.5**	**3.5**
**Compensatory payment (EUR ha-1)**	**706.0**	**163.5 (J culture in EA)***	**178.4 (R culture in EA)***	**-**	**-**
**Payment for SAPS, Greening, Top-Up**	**226.3**	**226.3**	**226.3**	**226.3**	**226.3**
**Revenues (EUR ha-1) – for sale of the commodities/wildflower strip establishment**	**0**	**934.0**	**154.0**	**1274.0**	**1643.3**
**Agrotechnical operation costs (EUR ha-1)**	**136.6**	**134.0**	**122.2**	**308.1**	**320.2**
**Direct material costs (EUR ha-1)**	**122.4**	**-**	**161.8**	**330.7**	**485.4**
**Total gross margin (EUR ha -1)**	**673**	**1190**	**274**	**861.5**	**1064.0**

Notes: J culture – other permanent culture, R culture – standard arable land, EA – ecological agriculture
